# How do placebos work?

**DOI:** 10.1080/20008198.2018.1533370

**Published:** 2018-10-25

**Authors:** Fabrizio Benedetti, Alessandro Piedimonte, Elisa Frisaldi

**Affiliations:** a Department of Neuroscience, University of Turin Medical School, Turin, Italy; b Plateau Rosà Laboratories, Plateau Rosà, Italy/Switzerland

Clinical trials of post-traumatic stress disorder (PTSD) show a high rate of placebo response, ranging from 19% to 62% (Ipser & Stein, 2012; Kelmendi et al., 2016; Sher, 2004). Despite these placebo responses across a number of clinical trials, nothing is known about their underlying mechanisms. In fact, the clinical trial setting does not allow us to differentiate between placebo effects due to psychological factors such as patients’ expectations and other phenomena such as spontaneous remission and regression to the mean. The recent explosion of placebo research and the shift in conceptualization of the placebo effect in several medical conditions, such as pain and motor disorders, makes us understand that their psychological and neurobiological underpinnings can be investigated in some detail. Indeed, what we have learned over the past few years is that different brain mechanisms are at work during the placebo response (Benedetti, Amanzio, Rosato, & Blanchard, 2011; Benedetti, Carlino, & Piedimonte, 2016; Wager & Atlas, 2015).

A placebo is usually defined as an inert substance with no pharmacological action, or as a sham physical intervention, although this definition is not complete, as placebos are made of many things, such as words, rituals, symbols, and meanings. Therefore, a placebo is not the fake treatment per se, be it pharmacological or not, but rather its administration within a complex psychosocial context. Indeed, a placebo is the whole ritual of the therapeutic act (Benedetti, 2014). The confusion about the words placebo and placebo effect comes from the different usage that they have for the clinical triallist and the neuroscientist/psychologist. The former is interested in any improvement that may take place in the group of patients who take the inert substance, and this improvement can be due to plenty of factors, such as spontaneous remission, regression to the mean, and patient’s expectations of benefit. By contrast, the neuroscientist is only interested in the improvement that derives from the patient’s expectations, namely, an active process occurring in the patient’s brain. Clinical trials are only aimed at establishing whether the patients who take the true treatment are better off than those who take the placebo, whereas the neurosciences want to understand what is going on in the patient’s brain when a placebo is given, i.e. when a therapeutic ritual is performed. By using this neuroscientific approach, the placebo effect represents an excellent model to understand how the human brain works (Benedetti, 2014; Wager & Atlas, 2015) and may have profound implications for both medical practice and clinical trials (Benedetti et al., 2016).

One of the most interesting and challenging aspects of placebo research is related to the emerging concept that placebos activate the same biochemical pathways that are activated by drugs (Benedetti, 2014; Benedetti et al., 2016), which represents quite an interesting challenge from both an evolutionary and a neurobiological perspective. Humans are endowed with endogenous systems that can be activated by verbally induced positive expectations, therapeutic rituals, healing symbols, and social interactions, and these systems include both endogenous opioids and endocannabinoids in placebo analgesia (Benedetti et al., 2011) and dopamine in Parkinson-related placebo responses (de la Fuente-Fernández et al., 2001). When morphine is administered, it binds to opioid receptors and inhibits pain transmission, but at the same time the ritual of its administration induces the activation of the same opioid receptors, involving a descending pain modulating network that goes from the cortex down to the spinal cord (Eippert, Bingel, et al., 2009; Eippert, Finsterbusch, Bingel, & Büchel, 2009). Similarly, when an anti-Parkinson dopaminergic drug is given it stimulates dopamine receptors, but at the same time the ritual of its administration activates the same dopamine receptors (de la Fuente-Fernández et al., 2001), along with substantial changes in neuronal activity in the basal ganglia (Benedetti et al., 2004). More recent findings indicate that the cyclooxygenase pathway can be affected by placebos as well (Benedetti, Durando, & Vighetti, 2014), thus suggesting an intricate set of mechanisms, including enzymatic activity, that can be activated by psychosocial stimuli, such as patients’ expectations of improvement and different therapeutic rituals.

It goes without saying that clear-cut differences between placebo and drugs do exist (Benedetti et al., 2016). First, as far as we know today, the duration of the effect of a drug is longer than that of a placebo. For example, the effect of the powerful anti-Parkinson drug apomorphine lasts on average much longer than a placebo. The mean duration of apomorphine is around 90 min, whereas the mean duration of the placebo effect is about 30 min. Secondly, the variability of the response is different, such that the clinical response is much more variable after placebo administration than after apomorphine. As far as the magnitude of the response is concerned, the effect following placebo administration can be as large as the effect following drug administration. For example, some good placebo responders may show a reduction in the Unified Parkinson’s Disease Rating Scale (UPDRS) of up to 50%, as also occurs for anti-Parkinson drugs. The placebo effect can be even larger in pain, where pain reduction can be of 5–6 points on a scale ranging from 0 = no pain to 10 = unbearable pain, as occurs in irritable bowel syndrome, where the analgesic response to a placebo has been found in a study to be even larger than that to lidocaine (Vase, Robinson, Verne, & Price, 2003). However, it is important to point out that only a small percentage of placebo responders may show such huge effects.

A crucial point in placebo research is the understanding of why some people respond whereas others do not. Different studies have addressed this important point and now this approach is paying dividends and bodes well for the future. Neuroimaging research has shown that prefrontal circuitry is always involved in placebo analgesia, in studies on chronic back pain (Hashmi et al., 2012), fibromyalgia (Schmidt-Wilcke et al., 2014), and chronic knee osteoarthritis (OA) (Tétreault et al., 2016). The right midfrontal gyrus (r-MFG) connectivity was specifically identified as a biomarker that can predict the placebo response in patients affected by OA (Tétreault et al., 2016). Activity in the r-MFG was observed to be related to decision making, memory, and planning processes, thus supporting the idea that placebo analgesia is driven by a complex top–down modulation. The amygdala, nucleus accumbens (NAc), and ventral striatum (VS) were also found to be regions closely connected to the medial prefrontal circuitry. Placebo analgesic treatments reliably reduce activity in the amygdala and increase activity in the NAc-VS region (Atlas & Wager, 2014). In particular, interindividual differences in NAc-VS seem to be important for identifying placebo responders and non-responders. Indeed, strong placebo analgesic effects are predicted by NAc-VS activity, including stronger placebo-related opioid and functional magnetic resonance imaging (fMRI) activity responses during pain, increased grey matter volume, and stronger fMRI responses in a reward pursuit task unrelated to pain (Wager & Atlas, 2015).

Beside these advances in neuroimaging, the pharmacological experimental approach has shown that placebo analgesic effects can be boosted using both vasopressin and oxytocin agonists. In particular, a randomized trial on healthy participants showed that vasopressin increases placebo analgesia significantly compared to no treatment, oxytocin, and saline, and these effects were found only in women. Moreover, women with both lower dispositional anxiety and cortisol levels showed the largest vasopressin-induced modulation of placebo effects, suggesting a moderating interplay between pre-existing psychological factors and cortisol changes (Colloca, Pine, Ernst, Miller, & Grillon, 2016).

Personality traits also contribute towards explaining a substantial proportion of the variance in placebo analgesic effects; placebo effects have been found to be related to optimism, suggestibility, empathy, and neuroticism, whereas nocebo effects have been linked to pessimism, anxiety, and catastrophizing (Corsi & Colloca, 2017). Attempts to describe the placebo responder or non-responder through a single personality trait may be limiting and, indeed, a transactional model of placebo responding, in which dispositional characteristics dynamically interact with environmental contingencies, was presented in 2015 (Darragh, Booth, & Consedine, 2015). The overlaps among the personality traits identified so far suggest that placebo responsiveness could be conceptualized in terms of a two-faceted construct consisting of an inward and an outward orientation. Persons with ‘inward orientation’ show a tendency to have an internal focus, or the ability to respond to suggestions regarding internal experience, whereas persons with ‘outward orientation’ are pervious to external inputs. Absorption, suggestibility, and acquiescence are personality traits that can be included in the first facet of placebo responsiveness, whereas resiliency, altruism, straightforwardness, optimism, extraversion, and dopamine-related traits belong to the second facet of placebo responsiveness. According to this transactional model, when a match between the type of individual and the nature of the contextual cues is missing, responding may not arise. Consequently, health practitioners could maximize the placebo component of any treatment or increase the chance of responding to treatment by tailoring clinical approaches based on how certain individuals respond to contextual treatment. In a recent experimental model of conditioning and heat thermal painful stimulation, the potential advantage of considering several personality factors was confirmed (Corsi & Colloca, 2017). Moreover, it was shown for the first time that expectations predict placebo and nocebo effects independently of personality factors. In fact, expectations were found to highly correlate with placebo and nocebo effects, whereas psychological factors per se did not influence levels of expectation of either reduction or increase in pain.

In light of this new knowledge about placebo effects, the crucial question is related to where (in which medical condition), when (in which circumstance, e.g. at home or in the hospital), and how (which mechanisms) placebos work. Accordingly, PTSD represents a neuropsychiatric disorder worthy of scientific investigation, from both a psychological and a neurobiological perspective. To do this, we should disentangle patients’ expectations from spontaneous remission and regression to the mean in well-conceived and well-designed PTSD clinical trials. Once the psychological component of placebo treatments for PTSD has been identified and well described, the psychobiological underpinnings should be investigated. For example, neuroscientific research should be aimed at assessing whether some endogenous systems, such as opioids and/or cannabinoids, are involved in the placebo response of PTSD.

Therefore, we need to change our perspective about placebo effects and conceptualize them in a different way, so that they can be considered as phenomena worthy of scientific inquiry. A better understanding of where, when, and how placebos work represents an important challenge for future research, which will surely lead to better medical practice and better interpretation of clinical trials. As we know virtually nothing about placebo effects in PTSD, this neuropsychiatric condition can be an excellent model to unravel new mechanisms and to answer some unanswered questions. The crucial starting point is the understanding of the biological underpinnings. We believe that medical practice and clinical trials, as well as human biology and neuroscience, will benefit from this new knowledge.
